# Diagnostic Assays for Crimean-Congo Hemorrhagic Fever 

**DOI:** 10.3201/eid1812.120710

**Published:** 2012-12

**Authors:** Jessica Vanhomwegen, Maria João Alves, Tatjana Avšič Županc, Silvia Bino, Sadegh Chinikar, Helen Karlberg, Gülay Korukluoğlu, Miša Korva, Masoud Mardani, Ali Mirazimi, Mehrdad Mousavi, Anna Papa, Ana Saksida, Batool Sharifi-Mood, Persofoni Sidira, Katerina Tsergouli, Roman Wölfel, Hervé Zeller, Philippe Dubois

**Affiliations:** Author affiliations: Institut Pasteur, Paris, France (J. Vanhomwegen, P. Dubois);; European Centre for Disease Control, Stockholm, Sweden (J. Vanhomwegen, H. Zeller);; National Institute of Health Dr. Ricardo Jorge, Lisbon, Portugal (M.J. Alves);; Faculty of Medicine, Ljubljana, Slovenia (T. Avšič Županc, M. Korva, A. Saksida);; Institute of Public Health, Tirana, Albania (S. Bino);; Pasteur Institute of Iran, Tehran, Iran (S. Chinikar);; Swedish Institute for Infectious Disease Control, Solna, Sweden (H. Karlberg, A. Mirazimi, M. Mousavi);; Refik Saydam National Public Health Agency, Ankara, Turkey (G. Korukluoğlu);; Shahid Beheshti University of Medical Sciences, Tehran, Iran (M. Mardani, B. Sharifi-Mood);; Aristotle University of Thessaloniki, Thessaloniki, Greece (A. Papa, P. Sidira, K. Tsergouli);; and Bundeswehr Institute of Microbiology, Munich, Germany (R. Wölfel)

**Keywords:** Crimean-Congo hemorrhagic fever, vector-borne infections, viruses, CCHFV, diagnostics, ELISA, IFA, qRT-PCR, multicenter, serology, molecular, diagnosis, reverse transcription PCR, PCR, immunofluorescent assay, tick-borne infections, CCHF

## Abstract

Crimean-Congo hemorrhagic fever (CCHF) is a highly contagious viral tick-borne disease with case-fatality rates as high as 50%. We describe a collaborative evaluation of the characteristics, performance, and on-site applicability of serologic and molecular assays for diagnosis of CCHF. We evaluated ELISA, immunofluorescence, quantitative reverse transcription PCR, and low-density macroarray assays for detection of CCHF virus using precharacterized archived patient serum samples. Compared with results of local, in-house methods, test sensitivities were 87.8%–93.9% for IgM serology, 80.4%–86.1% for IgG serology, and 79.6%–83.3% for genome detection. Specificity was excellent for all assays; molecular test results were influenced by patient country of origin. Our findings demonstrate that well-characterized, reliable tools are available for CCHF diagnosis and surveillance. The on-site use of such assays by health laboratories would greatly diminish the time, costs, and risks posed by the handling, packaging, and shipping of highly infectious biologic material.

## Abstract 2

On-site testing would diminish time, costs, and risks involved in handling of highly infectious materials.

Crimean-Congo hemorrhagic fever (CCHF) is a tick-borne zoonotic disease caused by a virus (CCHFV) belonging to the *Nairovirus* genus (*1*). The disease is asymptomatic in infected animals but can develop into severe illness in humans, with case-fatality rates as high as 50% in some outbreaks (*2*,*3*). The incubation period is typically 3–7 days, with sudden onset of myalgia, headache, and fever that can develop into a severe hemorrhagic syndrome (*4*,*5*). CCHFV is transmitted by tick bite (from mainly *Hyalomma* spp. ticks) or by contact with blood or tissues from infected livestock or patients with CCHF (*2*,*6*).

Sporadic cases of CCHF and community and nosocomial outbreaks have been increasingly reported, and the disease’s geographic distribution is the most extensive among tick-borne diseases. Currently, CCHFV is enzootic in southeastern Europe (Bulgaria, Albania, Kosovo, and Greece), southern Russia, and several countries in the Middle East, Africa, and Asia (*7*–*9*). Given the abundance of vectors, potential hosts, favorable climate and ecology, and intensified human travel, emergence and rapid establishment of new CCHF foci in other countries are substantial risks (*10*). Emergence or reemergence of CCHF poses a serious public health threat because it is highly contagious and highly lethal, has the potential to cause nosocomial infection, and is difficult to treat, prevent, and control. In addition to enhanced surveillance and development of therapeutics, access to early, sensitive, and specific laboratory diagnosis is a key factor in increasing preparedness in Europe and other countries at risk (*11*–*13*).

Although viral isolation is the standard for CCHF diagnosis, because it has to be done in high-containment biosafety level 4 facilities, the number of laboratories that can perform this technique is limited. Moreover, because cell cultures lack sensitivity and usually only detect the relatively high viremia level encountered during the first 5 days of illness, viral isolation is not without error or uncertainty. As a consequence, reference laboratories have been using the best available practicable methods to determine the presence or absence of infection (*11*). These methods include conventional and real-time quantitative reverse transcription PCR (RT-PCR and qRT-PCR) for detection of the viral genome (*14*–*18*) and indirect immunofluorescence assays (IFAs) or ELISAs for detection of specific IgM and IgG antibodies (*19*–*22*). No consensus on the most efficient molecular and serologic testing method has been reached.

In this context, a working group of experts from reference laboratories was constituted under the initiative of the European Network for Diagnostics of Imported Viral Diseases to take part in a multicenter study of CCHF diagnostic tests. The aim of this study was to evaluate and compare the performance of, and review the operational characteristics of, available CCHF diagnostic tests by using panels of well-characterized, archived serum samples from patients from geographically diverse settings.

## Materials and Methods

### Study Participants and Diagnostic Tools

Experts from 5 institutions participated in this study: Bundeswehr Institute of Microbiology, Munich, Germany; Department of Microbiology, Aristotle University of Thessaloniki, Thessaloniki, Greece; Center for Vectors and Infectious Diseases Research, National Institute of Health, Águas de Moura, Portugal; Institute of Microbiology and Immunology, Medical Faculty, Ljubljana, Slovenia; and Center for Microbiological Preparedness, Swedish Institute for Infectious Disease Control, Solna, Sweden. Diagnostic methods that could be performed in standard laboratory facilities were selected on the basis of a systematic review of the literature and the experiences of the members of the working group. During April 2010, an extensive search of available CCHF diagnostic tools was performed by using both generic (Google) and scientific (PubMed) Internet-based search engines. To meet the selection criteria, assays had to be commercially available or in the prerelease phase at the time of our assessment (or have quality assessed reagents and well-defined protocols for noncommercial assays); yield early and rapid results (within 5 hours); not require the purchase of specific equipment; and have demonstrated sufficient scope to detect diverse CCHFV variants or antibodies. The reporting of results was conducted according to Standards for Reporting of Diagnostic Accuracy criteria (www.stard-statement.org; Technical Appendix Table 1).

**Table 1:** Reference methods used by the reference laboratories that participated in evaluation of serologic and molecular assays for diagnosis of CCHF

Laboratory	Reference IgM serologic tests	Reference IgG serologic tests	Reference molecular tests
1	In-house IFA (CCHFV-infected cells)	In-house IFA (CCHFV-infected cells)	Nested RT-PCR (*23*) and qRT-PCR (*24*)
2	In-house IgM capture ELISA (CCHFV strain IbAr10200 antigen)	In-house sandwich ELISA (CCHFV strain IbAr10200 antigen)	Nested RT-PCR (*25*) and qRT-PCR (*17*)
3	In-house IFA (CCHFV strain ArD39554 infected cells)	In-house IFA (CCHF strain ArD39554 infected cells)	qRT-PCR (*18*)
4	In-house IFA (CCHFV infected cells)	In-house IFA (CCHF infected cells)	qRT-PCR (*18*)
5	Testing performed by external reference laboratory	Testing performed by external reference laboratory	qRT-PCR (*18*)

*CCHF, Crimean-Congo hemorrhagic fever; IFA, immunofluorescence assay; CCHFV, CCHF virus; RT-PCR, reverse transcription PCR; qRT-PCR, quantitative RT-PCR.

### Patient Status Definition and Samples

Because no reference test for CCHV is universally accepted, patients with clinically suspected CCHF were confirmed on the basis of results of serologic and molecular diagnostic tests that were in use in the CCHF reference laboratories at the time of the study (Table 1). These cases were defined by the either positive rule: detection of CCHFV genome or CCHFV-specific IgM, IgG, or both, during either the acute or convalescent phase of the disease. Each participant in the working group contributed a panel of archived serum samples that had tested positive for CCHFV by IgM, IgG, or both, and a panel of archived serum RNA extracts from which CCHFV genome had been detected; samples were collected from patients with laboratory-confirmed CCHF infection. Negative controls were samples from healthy persons who originated from disease-endemic or at-risk areas (e.g., blood donors) and samples from febrile patients with a diagnosis of 1 of the conditions that can produce symptoms similar to those of CCHFV infection (e.g., hemorrhagic fever with renal syndrome, leptospirosis, West Nile fever, chikungunya). All samples were included in the study with the consent of the patients.

### Assay Methodology and Data Collection

Tests were performed according to the manufacturers’ instructions or according to validated protocols provided by the developer. Working group members tested the selected assays in duplicate on their respective sample panels within their facilities. Results of qualitative assays (IFAs and low-cost, low-density [LCD] arrays) were read by 2 independent readers. Results were collected at the end of each testing session by using a standard report datasheet and combined into a final database.

### Data Analysis

Results obtained for each selected diagnostic tool were compared in a 2×2 table with results from the reference in-house diagnostic to estimate indices of sensitivity, specificity, and corresponding 95% CIs. In addition, test results were compared with the results of a composite reference standard (positive if detection of specific CCHFV genome or specific CCHF IgM or IgG antibodies by in-house reference methods; negative otherwise) to confirm the specificity estimates and corresponding 95% CIs. Statistical analysis was performed in STATA/SE version 12.0 software (StataCorp, College Station, TX, USA). CIs were calculated by using binomial exact methods. A univariate analysis was conducted by using the Fisher exact test to identify factors influencing test sensitivity (i.e., patient country of origin, severity of disease, number of days after illness onset that sample was collected, and sample storage time before testing); p<0.05 was considered significant. A multinomial exact logistic regression analysis was performed to identify independent factors influencing sensitivity, including all variables associated with sensitivity in the univariate analysis (p<0.1). Data on operational characteristics of each test (i.e., ease-of-use, level of staff training required, time, ease of interpretation) were gathered through a questionnaire. Tests were scored on operational characteristics.

## Results

### Selected Diagnostic Methods

Six diagnostic assays met the criteria for inclusion in the study. For specific CCHF serodiagnosis, a commercial IgM and IgG ELISA (Vector-Best, Novosibirsk, Russia) and a commercial IgM and IgG IFA (Euroimmun, Luebeck, Germany) were selected. For detection of the CCHFV genome, a commercial real-time RT-PCR (Altona-Diagnostics, Hamburg, Germany) and a low-cost, low-density macroarray (*26*) were used. Characteristics of the selected tests are shown in Table 2. After selection, assays were purchased directly from the manufacturers and shipped according to their instructions to the working group members by express delivery.

**Table 2:** Characteristics of selected assays compared in study of CCHF diagnostic tools

Characteristics	IgM ELISA	IgG ELISA		IgM IFA	IgG IFA	qRT-PCR	LCD array
Assay (manufacturer, location or reference)	VectoCrimea-CHF ELISA (Vector-Best, Novosibirsk, Russia)			Crimean-Congo Fever Mosaic 2 IFA (Euroimmun, Luebeck, Germany)		RealStar CCHFV RT-PCR Kit 1.2 (Altona-Diagnostics, Hamburg, Germany)	CCHF2006 1.5 LCD Kit (*26*)
Reference no.	D-5054		D-5056	FI 279a-1010-2M	FI 279a-1010-2G	181203	NA
Target	CCHFV-specific IgM		CCHFV-specific IgG	CCHFV-specific IgM	CCHFV-specific IgG	CCHFV S segment	CCHFV S segment
Shelf life, mo	9		9	18	18	12	Unknown
Storage temperature, °C	2 to 8		2 to 8	2 to 8	2 to 8	−15 to −25	2 to 8, −20
Quoted accuracy, %							
Sensitivity	100		100	97.2	89.5	Unknown	100
Specificity	100		100	97.5	100	Unknown	100
Sample type	Serum, plasma		Serum, plasma	Serum, plasma	Serum, plasma	RNA extract from serum or blood	RNA extract from serum or blood
Sample volume, µL	10		10	5	5	10	10
Minimum kit format (no. reactions)	12 strips × 8 tests (96)		12 strips × 8 tests (96)	10 slides × 5 tests (50)	10 slides × 5 tests (50)	8 tubes × 12 tests (96)	4 slides × 8 tests (32)
Price, Euros†							
Per kit	139.2		139.2	326	326	1,200	Unknown
Per reaction	1.45		1.45	6.51	6.51	12.50	Unknown
Estimated run time, min	175		175	70	70	58	175

*CCHFV, Crimean-Congo hemorrhagic fever virus; IFA, immunofluorescence assay; qRT-PCR, quantitative reverse transcription PCR; LCD, low-cost, low-density; NA, not applicable; S segment, small segment. †Does not include shipping costs.

### Characteristics of Study Population and Sample Panels

The serum panel constituted for the evaluation of the serologic tests consisted of 66 stored serum samples from acute-phase CCHF patients (those who recovered or died) and patients with confirmed CCHF diagnosis who had an early recovery; 32 samples from febrile patients who had symptoms compatible with CCHFV infection; and 41 samples from healthy persons. Molecular tests were evaluated by using a panel of RNA extracts from acute-phase patient serum samples: 54 samples from patients with confirmed CCHF diagnosis, 16 samples from febrile patients who had symptoms compatible with CCHFV infection, and 5 samples from healthy persons. Characteristics of the patient population and the sample panels are shown in Table 3. Confirmed CCHF case-patients originated from Iran, Kosovo, Albania, Turkey, and sub-Saharan Africa; most had moderate CCHF. Patients with symptoms compatible with CCHF infection included patients who had a diagnosis of leptospirosis, chikungunya fever, hemorrhagic fever with renal syndrome (HFRS), Q fever, tularemia, brucellosis, and West Nile encephalitis. Serum samples were collected 5–49 days after onset of symptoms; RNA extracts were obtained from serum samples collected 2–14 days after onset of symptoms. Storage time until testing ranged from 1 to 23 years for serum samples and 1 to 4 years for RNA extracts.

**Table 3:** Patient characteristics and sample storage information for samples tested for CCHFV

Characteristics	Sample panel 1, serology, no. (%)		Sample panel 2, genome detection, no. (%)

CCHFV positive, n = 66	CCHFV negative, n = 73	CCHFV positive, n = 54	CCHFV negative, n = 21
Conditions					
CCHF	66 (100.0)	0		54 (100.0)	0
Brucellosis	0	2 (2.7)		0	2 (9.5)
Chikungunya	0	1 (1.4)		0	1 (4.8)
HFRS	0	13 (17.8)		0	7 (33.3)
Leptospirosis	0	13 (17.8)		0	3 (14.3)
Q fever	0	1 (1.4)		0	1 (4.8)
Tularemia	0	1 (1.4)		0	1 (4.8)
West Nile fever	0	1 (1.4)		0	1 (4.8)
Healthy or non-CCHF	0	41 (56.2)		0	5 (23.8)
Patient country of origin					
Albania	9 (13.6)	0		8 (14.8)	0
Germany	0	23 (31.5)		0	7 (33.3)
Greece	0	9 (12.3)		0	9 (42.9)
Iran	32 (48.5)	0		31 (57.4)	0
Kosovo	21 (31.8)	20 (27.4)		7 (13.0)	4 (19.0)
Portugal	0	20 (27.4)		0	0
Sub-Saharan Africa	4 (6.1)	0		0	0
Turkey	0	1 (1.4)		8 (14.8)	1 (4.8)
CCHF disease severity					
Moderate	49 (74.2)	0		37 (68.5)	0
Severe	11 (16.7)	0		2 (3.7)	0
Fatal	3 (4.5)	0		6 (11.1)	0
Asymptomatic	3 (4.5)	0		7 (13.0)	0
Unknown	0	0		2 (3.7)	0
Length of illness, d					
<15 d	46 (69.7)	0		43 (79.6)	0
≥15 d	12 (18.2)	0		0	0
Asymptomatic	3 (4.5)	0		7 (13.0)	0
Unknown	5 (7.6)	0		4 (7.4)	0
Sample storage time, y					
<10	49 (74.2)	64 (87.7)		54 (100.0)	10 (47.6)
≥10	17 (25.8)	0		0	2 (9.5)
Unknown	0	9 (12.3)		0	9 (42.9)
Sample storage temperature, °C					
−80	34 (51.5)	49 (67.1)		9 (16.7)	4 (19.1)
−70	32 (48.5)	0		45 (83.3)	9 (42.9)
−20	0	24 (32.9)		0	8 (38.1)

*CCHF, Crimean-Congo hemorrhagic fever; CCHFV, CCHF virus; HFRS, hemorrhagic fever with renal syndrome.

### Performances of Selected CCHF IgM Serology Assays

A total of 138 and 90 samples from the collected patient serum panels were tested for CCHFV-specific IgM by the Vector-Best ELISA and the Euroimmun IFA, respectively. Because of limited sample amounts, IFA could not be performed on all collected samples. When compared with the reference IgM serology tests, the sensitivity of the IgM ELISA ranged from 75.0% to 100.0% for different laboratories, with an overall sensitivity of 87.8% (95% CI 78.6%–96.9%). For the IgM IFA, sensitivity ranged from 75.0% to 100.0%, with an overall sensitivity of 93.9% (95% CI 85.8%–100.0%). Overall specificity was estimated to be 98.9% (95% CI 96.7%–100.0%) for the IgM ELISA and 100% for the IgM IFA (Table 4). When a composite reference standard (described in the Methods section) was used as reference, the observed specificity was 100% for both tests.

**Table 4:** Overall performance of assays compared in study of CCHF diagnostic tools

Parameter	IgM serology			IgG serology			Genome detection	
	ELISA	IFA		ELISA	IFA		qRT-PCR	LCD array
No. samples tested	138	90		137	92		71	70
No. true positive	43	31		41	31		39	40
No. false negative	6	2		10	5		10	8
No. true negative	88	57		86	56		21	21
No. false positive	1	0		0	0		1	1
Sensitivity, % (95% CI)	87.8 (75.2–95.3)	93.9 (79.8–99.3)		80.4 (66.9–90.2)	86.1 (70.5–95.3)		79.6 (65.7–89.8)	83.3 (69.8–92.5)
Specificity, % (95% CI)	98.9 (93.9–100.0)	100.0 (93.7–100.0)†		100.0 (95.8–100.0)	100.0 (93.6–100.0)		95.5 (77.2–99.9)	95.5 (77.2–99.9)

*CCHF, Crimean-Congo hemorrhagic fever; IFA, immunofluorescent assay; qRT-PCR, quantitative reverse transcription PCR; LCD, low-cost, low-density. †One-sided 95% CI.

### Performances of Selected CCHF IgG Serology Assays

A total of 137 and 92 samples from the collected patient serum panel were tested for CCHFV-specific IgG by the Vector-Best ELISA and the Euroimmun IFA, respectively. When compared with the reference IgG serology tests, the estimated sensitivity for the IgG ELISA ranged from 75.0% to 100.0%, with an overall sensitivity of 80.4% (95% CI 69.5%–91.3%). For the IgG IFA, sensitivity ranged from 40.0% to 100.0%, with an overall sensitivity of 86.1% (95% CI 74.8%–97.4%). Specificity was estimated to be 100% for both assays (Table 4).

### Performances of Selected CCHF Molecular Assays

A total of 71 and 70 samples, respectively, from the collected panel of serum RNA extracts were tested for the presence of the CCHF genome by the Altona-Diagnostics CCHFV qRT-PCR and the CCHF LCD array. When compared with the results of the reference genome detection methods, sensitivity ranged from 42.9% to 100%, with an overall sensitivity of 79.6% (95% CI 68.3%–90.9%) for the qRT-PCR and from 25.0% to 100% with an overall sensitivity of 83.3% (95% CI 72.8%–95.5%) for the LCD array. Both assays demonstrated a specificity of 95.5% (95% CI 90.6%–100%) (Table 4), which increased to 100% when the results of a composite reference standard were used as reference.

### Factors Influencing Diagnostic Sensitivity

The influence of several patient and sample characteristics on the sensitivity of the selected assays was analyzed by univariate analysis (Technical Appendix Table 2). The country of origin of the patient was found to be significantly associated with the sensitivity of the IgG IFA (p = 0.02), the qRT-PCR (p<0.001), and the LCD array (p = 0.02). However, after multivariate analysis, this association only remained significant for the qRT-PCR assay (p<0.001). In particular, the qRT-PCR was found to be less sensitive for samples from patients originating from Turkey (adjusted odds ratio [OR] 0.04, 95% CI 0.00–0.87) and from Albania (adjusted OR 0.02, 95% CI 0.00–0.16).

### Operational Characteristics of the CCHF Diagnostics

Scores for operational characteristics are summarized in Table 5. The ELISA test obtained a higher overall score (8.5/10) compared with the IFA (6.7/10). The IFA scored lowest in the ease of interpretation of results (1.3/2) and in the requirement for specific technical training (0.3/1). The observed scores for molecular tests were within the same range (6.0–6.3/10). Both molecular assays demonstrated low scores for technical complexity (1.3–1.5/2) and training requirements for equipment and technique (0.3–0.5/1).

**Table 5:** Operational characteristics of selected CCHF diagnostic assays*

Operational characteristic	Mean score			
	VectoCrimea-CHF ELISA†	Crimean-Congo Fever Mosaic 2 IFA‡	RealStar CCHFV RT-PCR Kit 1.2§	CCHF^2006^ 1.5 LCD Kit (*26*)
Equipment¶				
Maintenance of equipment required	0.8	0.3	0.3	0.3
Training for equipment required	0.8	0.7	0.3	0.5
Additional equipment required	0.8	1.0	0.5	0.8
Technique				
Clarity of instructions¶	1.0	0.7	1.0	1.0
Technical training required¶	0.8	0.3	0.3	0.3
Technical complexity#	2.0	1.7	1.5	1.3
Interpretation				
Training required for result interpretation¶	1.0	0.7	0.8	0.8
Ease of interpretation of results#	1.5	1.3	1.5	1.5
Total score	8.5/10	6.7/10	6.0/10	6.3/10

*CCHF, Crimean-Congo hemorrhagic fever; IFA, immunofluorescent assay; CCHFV, CCHF virus; RT-PCR, reverse transcription PCR; LCD, low-cost, low-density. †Vector-Best, Novosibirsk, Russia. ‡Euroimmun, Luebeck, Germany. §Altona-Diagnostics, Hamburg, Germany. ¶A score of 1 was attributed when instructions were sufficiently clear or when no specific training, no additional equipment, or no regular equipment maintenance were necessary. #Score was attributed according to the degree of simplicity of the technique or interpretation: 2 if it was considered easy, 1 if it was considered acceptable, and 0 if it was considered difficult.

## Discussion

A number of published studies have described major epidemics, community and nosocomial outbreaks, and the ecology of CCHF (*4*,*7*,*8*,*27*–*30*). These reports have shown that a distinct epidemiologic situation can arise in regions where the virus is endemic but also that new foci can emerge (*10*,*31*). The World Health Organization has listed CCHF among the emerging diseases for which control and prevention measures should be renewed and intensified (*13*,*32*). In addition, an assessment of the importance and magnitude of vector-borne diseases initiated by the European Centre for Disease Prevention and Control identified CCHF as a priority disease for the European Union (*33*).

A strong laboratory capacity, in particular standardized approaches for diagnostic methods and assay validation, has been identified as a short-term priority in CCHF-endemic areas and regions where CCHFV could be expected to circulate (*11*,*12*). The aim of this study was to identify and evaluate easily available, simple-to-perform CCHF diagnostic methods considered most suitable for widespread use in CCHF-endemic areas and countries at risk.

We assessed the performances of 2 commercially available IgM and IgG serologic tests, the Vector-Best CCHF ELISA and the Euroimmun CCHF IFA, and 2 assays for viral genome detection, the Altona-diagnostics CCHF qRT-PCR and a CCHF LCD array. The IgM and IgG ELISAs showed a sensitivity of 88% and 80%, respectively, lower than the numbers given by the manufacturer. These assays were validated by the manufacturer by using serum panels from CCHF cases originating from southwestern Russia (S. Suchkov, pers. comm.). Therefore, lower sensitivity for certain serum samples tested in this study may reflect antigenic variation among CCHFVs circulating in other countries.

Observed sensitivities of the IgM and IgG IFA were 93.9% and 86.1%, respectively. Although these estimates are higher than those observed for the IgM and IgG ELISAs, these results may have had a sampling bias because not all serum samples tested by ELISA could be tested by IFA. This bias was, however, minimized, because the tested serum panel included 15/16 false-negative samples observed for the ELISAs.

The sensitivity of the selected molecular assays was found to be more modest (79.6% for qRT-PCR and 83.3% for LCD array) than for serologic methods and to be associated with the patient country of origin. This result is consistent with the finding that the application of molecular assays in different settings is hampered by the high diversity of the CCHFV genomes, whereas serologic methods can have a broader use due to cross-reactivities. In particular, the qRT-PCR seems to be less sensitive for patients originating from the Balkans region and Turkey than for patients from other countries compared with in-house reference molecular methods. The in-house methods developed by reference laboratories are optimized for detection of strains circulating in that area, which may result in a lower detection limit when compared with methods that cover a broader spectrum. However, other factors, such as RNA degradation due to freezing and thawing cycles or inhibition of PCR reactions because of inhibitory compounds in the samples, may have contributed to decreased sensitivity of molecular methods compared with serologic methods.

The observed specificity was excellent for all assays, ranging from 95.5% to 100% when compared with the reference method and equal to 100% when compared with a composite reference standard. However, predictive positive and negative values for the different assays could not be calculated because precise prevalence data are not available for most CCHF-endemic areas, and these data cannot be predicted for areas where the virus could emerge.

The CCHF IFA demonstrated a higher complexity in equipment, technique, and interpretation. However, the interpretation of fluorescence patterns may enable trained users to differentiate positive from cross-reactive serum samples, whereas such false positives may not be avoided with the ELISA. The operational characteristics of the molecular assays were comparable. Both methods required regular equipment maintenance and specific training for appropriate use of the equipment; however, this was more apparent for the real-time qRT-PCR method. The LCD array technique was considered to be complex but acceptable. Training for technique and result interpretation was recommended for each method.

All participating laboratories are reference centers for CCHF laboratory diagnosis and surveillance in their respective countries, and some are World Health Organization collaborating centers. The protocols from reference methods in use at each site have been extensively validated previously (*16**–**20**,**24*). In addition, the laboratories participated in a recent international external quality assessment (*34*). Therefore, local conditions at the participating sites and validity of reference methods were considered comparable.

The multicenter design of this study enabled the testing of a large sample size, representative of >1 population, so findings could be more generally applicable. In addition, sample panels were constituted without any selection for disease severity, and negative controls included not only healthy patients but also patients who had a wide range of other conditions, thereby avoiding inflated estimates of diagnostic accuracy.

Our study has some limitations. Because archived samples were used for the study, specimen quality could have been affected; however, statistical analysis demonstrated that sample storage time and temperature did not influence sensitivity. Also, the use of different sample panels for serologic and molecular testing did not enable calculation of the added value of combining the serologic and molecular CCHF diagnostic methods evaluated.

The results of this study give additional guidance on the type of CCHF diagnostic tools that could be used in different contexts. During a large outbreak, easily interpretable tests for simultaneous analysis of numerous samples, such as the ELISA and real-time qRT-PCR, might be considered useful tools to identify CCHF cases. Methods available in smaller format size and demonstrating a long shelf life, such as the IFA and LCD array, could be used to identify sporadic cases or to confirm single cases as part of a larger outbreak.

This study demonstrates that efficient, well-characterized serologic and molecular assays and protocols are available for CCHF diagnosis. The on-site use of such assays by outbreak assistance laboratories would greatly diminish the risks posed by the handling, packaging, and shipping of highly infectious samples. Moreover, acquiring diagnostic reagents would be more time- and cost-effective for laboratories than would the organization of compliant packaging and shipment of hazardous biologic material to reference laboratories abroad. Nevertheless, laboratory personnel should receive the appropriate training to perform the different assays (e.g., during international workshops or network meetings). Collaborative evaluations of diagnostic methods remain essential to guide decision-making, especially with emerging diseases, where a standard is frequently missing, and laboratory expertise is rare.

Technical Appendix Checklist for reporting of studies of diagnostic accuracy and univariate analysis of factors influencing the sensitivity of Crimean-Congo hemorrhagic fever diagnostic assays.
